# Carbon-oxygen surface formation enhances secondary electron yield in Cu, Ag and Au

**DOI:** 10.1038/s41598-022-19924-9

**Published:** 2022-09-22

**Authors:** M. Brown, L. Diaz, A. Aslan, M. Sanati, S. Portillo, E. Schamiloglu, R. P. Joshi

**Affiliations:** 1 Department of Electrical and Computer Engineering, Texas Tech University, Lubbock, TX 79409 USA; 2 Department of Physics and Astronomy, Texas Tech University, Lubbock, TX 79409 USA; 3 Department of Electrical and Computer Engineering, University of New Mexico, Albuquerque, NM 87131 USA

**Keywords:** Engineering, Materials science, Physics

## Abstract

First-principles calculations coupled with Monte Carlo simulations are used to probe the role of a surface CO monolayer formation on secondary electron emission (SEE) from Cu, Ag, and Au (110) materials. It is shown that formation of such a layer increases the secondary electron emission in all systems. Analysis of calculated total density of states (TDOS) in Cu, Ag, and Au, and partial density of states (PDOS) of C and O confirm the formation of a covalent type bonding between C and O atoms. It is shown that such a bond modifies the TDOS and extended it to lower energies, which is then responsible for an increase in the probability density of secondary electron generation. Furthermore, a reduction in inelastic mean free path is predicted for all systems. Our predicted results for the secondary electron yield (SEY) compare very favorably with experimental data in all three materials, and exhibit increases in SEY. This is seen to occur despite increases in the work function for Cu, Ag, and Au. The present analysis can be extended to other absorbates and gas atoms at the surface, and such analyses will be present elsewhere.

## Introduction

Bombardment of materials by incident energetic ions, neutral atoms, and electrons can lead to the emission of secondary electrons. Secondary electron emission (SEE) was first discovered by Austin and Starke^1^, and since then, experimental^2–4^, theoretical^5–7^, and computational^8,9^ studies have contributed to an ever-increasing database for numerous materials. In metals, energetic primary electron knock-on collisions initiate cascades of momentum and energy transfer events involving the free-electron gas within the solid. A fraction of the primary incident electrons is elastically reflected, while the remainder penetrate and share their energy with the host material, thus creating energetic secondary electrons that can continue moving while undergoing additional collisions. As these secondaries penetrate deeper, they multiply and continue losing energy until they either rejoin the sea of conduction electrons, or reach the surface following a series of continuous scattering events. In the latter case, the electrons could emerge from the surface of the material provided they had sufficient energy to transcend the surface potential barrier. Hence, the surface condition can be expected to have an important influence on secondary electron emission. For example, the possible presence of surface absorbates including ambient gas atoms, or the formation of a thin oxide-layer at the emitting surface could conceivably alter the emission characteristics.

Secondary electron emission from solids has long been of interest for the understanding of electron beam–solid interactions^10,11^, in connection with possible effects on electronic device fabrication and lithography^12,13^, for detailed understanding of surface treatments^14^ and even in applications to medical technology^15^. While SEE is a fundamental aspect of electron-solid interaction, it is also important for the development of devices such as electron multipliers and electron microscopes^16,17^, and for RF/microwave generation^18–20^. While SEE may be advantageous for electron multipliers and electron microscopes, it is certainly detrimental to device performance in the vacuum electronics arena and for RF/microwave generation. The presence of SEE can alter the sheath potential and adversely affect the performance of high power devices^21^. Some drawbacks of SEE include the unstable erosion of material surfaces, multipactor^22–25^, performance degradation in rf accelerators, microwave components, and satellite communication systems due to the introduction of effects such as the electron cloud^26–28^ and electrostatic discharges^29,30^. Other possible deleterious effects include reductions in efficiencies of high power microwave devices, detuning in RF cavities and amplifiers, ionization breakdown, generation of undesirable harmonics, increases in the noise level, and local enhancements in electrode temperatures that can trigger outgassing possibly leading to gaseous discharges^31–36^.

In view of the above-mentioned adverse effects, suppression of secondary electron yield (SEY) has become an important goal from the standpoint of improving system stability and mitigating the destructive outcomes. A number of approaches have been tried and tested, including the use of artificially roughened surfaces^37–39^, geometric modifications^40^, the use of micro-porous array structures^30,41,42^, along with SEY lowering in the presence of a magnetic field^43,44^. It has been shown that for a deeply roughened surface, the secondary electrons excited by primary electrons may be trapped through collisions with the side walls of the gaps or pores distributed on the surface, thereby yielding a lower surface SEY. It may be mentioned for completeness that other reports of SEY suppression have included the use of copper oxide nanowires^45^, free-standing velvet whiskers^7^, graphite coatings^46–48^, and even surface laser ablation or laser-formed surface texturing^49–53^. A drawback to using coatings for SEY suppression is their susceptibility to aging and degradation. Other persistent issues, in general, include the inability to uniformly and strongly suppress SEE for a wide range of incident energies.

It is well known that SEY for materials depends on their surface composition and morphology^54–57^. Copper is a common electrode material for which SEY characteristics have been studied. Advantages of copper include accessibility and convenience in machining with relatively low loss and production of smooth uniformly textured surfaces. On the other hand, Ag and Au (though members of the same group in the periodic table) remain relatively unexplored, given their higher costs and inability to withstand rugged environments. All three metals, however, share similarities^58^ as they all belong to the same International Union of Pure and Applied Chemistry (IUPAC) group 11 of the periodic table. Hence, any theoretical analysis of a material property such as the SEY for copper, should perhaps also be applied in an extended manner to the evaluation in Au and Ag materials as well for consistency.

It is well known that the presence of the surface contaminants can increase or decrease the SEY significantly^59^. For example, as received samples of Cu, Ag, and Au that are not subjected to cleaning or any artificial treatment have SEY of about 200%^55,60,61^, while the values obtained from the corresponding cleaned sample surfaces have been found to be much lower at around 150%^55,60,61^. Since materials are exposed to atmosphere, surface contaminants could include water, carbon compounds, oxygen, nitrogen, and argon. Among all the contaminants, C and O have been reported to be present at the highest concentrations^55^. Furthermore, in numerous reports, removal of C and O from the samples has been seen to significantly reduce the SEY of the processed system^55,60,61^. However, there are also several experimental instances where carbonization or oxidization of metallic surface appear to lead to a reduction in the SEY of clean surfaces^55,62^. The overall situation thus appears somewhat murky and unclear. The extent of C or O if present at surfaces, would likely be very small (e.g., a few monolayers in extent at best), and insignificantly smaller than the electronic mean free paths. Hence, their influence on electron scattering or energy loss over the surface regions can be expected to be inconsequential in affecting the overall SEY characteristics. It is far more likely that their chemical interaction with the host material may be leading to changes in the workfunction, or local density of states, or bonding strengths that could then be the reason for observed SEY shifts If so, surface processing would certainly affect SEE.

Thus, the aim of this work is to use appropriate simulations to investigate the inherent physics and disentangle the role of each chemical surface component in influencing the SEY values. For concreteness and brevity, the analysis has been applied here for copper, gold and silver materials, though the method is general and would allow the investigation of other emitter materials and/or surface absorbates as well. While copper might have been sufficient for a numerical study, Au and Ag were additionally included here for a broader evaluation as these elements all belong to the same IUPAC group. The role of surface conditioning and the presence of the C and O contaminants is clarified. In the process, possible routes for developing inherently low-SEE emitters can then naturally emerge, though extensions to other materials and contaminants will be discussed elsewhere.

While existing literature on the relation between surface composition and SEY is substantial, much of it has been experimental or based on macroscopic or empirical analysis. Studies of a more fundamental nature, from electronic structure point of view, are relatively scarce. Furthermore, the role of the surface and any absorbates that may be present, has been ignored in most considerations and analyses. It is important to fill this gap in knowledge, as it will lead to a deeper understanding and better control over SEE, especially since the physics at the surface and the surrounding monolayers dictates the eventual outcomes. For instance, absorbates could alter the work function^63–66^, produce three-dimensional variations in density of states to affect the inherent source function, possibly cause lateral WF variations^67–69^.

Studying the formation of CO monolayer and its effect on physical properties like work function, inelastic mean free path, and secondary electronic emission are the subjects of this study. To better understand the general role of CO, we studied its formation in several systems (Cu, Ag, and Au) where experimental data is available for comparison. The studies presented here are carried out based on a combined first principles and Monte Carlo method. The results of first principles calculation of parameters such as the work functions, total density of states, frequency- and wavevector-dependent permitivities are necessary for obtaining the inelastic mean free path (IMFP), stopping power (SP), and SEY values based on Penn’s theory^70^. The electron emission process itself is simulated by including the associated transport, inherent scattering and exchanges of energy and momentum within the target, with eventual escape of electrons over the work function barrier at the surface. Monte Carlo simulations are used to model electron transport, taking account of elastic scattering based on the Mott theory, and inelastic collisions based on energy-dependent energy loss function and mean free paths^71,72^. The present MC scheme naturally builds in cascade effects associated with secondary and tertiary electron generation.

## Details of Calculations

### A. First-Principles

Here we apply a coupled simulation scheme that uses density function theory (DFT) for evaluation of the requisite material parameters, with a stochastic Monte Carlo (MC) method for treating the electron transport within the host material. Using the first-principles DFT package VASP, the necessary input parameters such as work functions, density of states, and permittivities required in the MC evaluations for the inelastic mean free path (IMFP), stopping power, and secondary electron were obtained. The VASP code uses a projector augmented-wave technique^73–76^. We employed the generalized gradient approximation (GGA) exchange–correlation functional as parameterized by Perdew, Burke, and Ernzerhof^77^ with a kinetic energy cutoff of 600 eV. The *k*-point integration was done by a modified tetrahedron method for the total and partial densities of states calculations^74,78^. The surface supercells consisted of ten host atoms spread over ten layers (vacuum region of about 16 Å), with a k-point sampling of 10 × 10 × 2 Monkhorst Pack mesh^79^. All surfaces were relaxed using their calculated bulk lattice constant since little change is expected^80,81^. The bulk lattice constants included 3.63 Å (3.61 Å), 4.16 Å (4.09 Å), and 4.17 Å (4.08 Å) for Cu, Ag, and Au, respectively, with their experimental values provided in parenthesis from Reference^82^. The top four layers were allowed to relax while the bottom six were held fixed. Finally, for all systems, DFT was used to calculate the frequency-dependent dielectric tensor^83^, an input parameter needed for obtaining the inelastic mean free paths and stopping power.

One of the required parameters of the Monte Carlo secondary electron emission simulation is the work function, which is dependent on the charge density of the surface. This requires calculations of the averaged potentials in the bulk and the surface. Using DFT, the three-dimensional potentials were obtained including the electrostatic, Hartree, and exchange–correlation potentials. When averaged along the x- and y- axes, these potentials ($V\left({\varvec{r}}\right)$) provide valuable information about the oscillations of the bulk and location of vacuum region along the z-direction. This averaging is known as the average planar potential ($\overline{V }(z)$) and is calculated using:

1 $$\overline{V }(z)=\frac{1}{S}\int V\left({\varvec{r}}\right)dxdy$$

where *S* is the area of the unit cell. Using the average planar potential, the work function was obtained using the following relation:

2 $$\varphi = {E}_{V} -{E}_{F}$$

where $\varphi$ is the work function, *E*_*V*_ is the electrostatic potential of the vacuum, and *E*_*F*_ is the Fermi level energy. The electrostatic potential of the vacuum or vacuum level is a region outside of the surface where an ejected electron no longer experiences electrostatic effects.

### B. Inelastic mean free path, stopping power, and monte carlo

Calculations of secondary electron emission are usually based on the well-known numerical Monte Carlo technique. This is a kinetic scheme in which incident electrons are treated as billiard balls, and the evolution of their motion through a host material is tracked based on Newtonian mechanics. The motion is peppered by a repetitive series of elastic and inelastic collisions based on computations of the energy dependent mean free path. The inelastic mean free path (IMFP) are given later in this section through Eqs. (4) and (7). At the end of each IMFP, an electron is assumed to undergo energy loss, with the magnitude being calculated from the stopping power ($\frac{dE}{dR}$) as discussed in later in connection with Eq. (5). Due to such collisions the energy angles and trajectories of electrons change, and secondaries are created from the energy exchange with a target electron. Details of the Monte Carlo implementation are well known (including our own treatment for nickel^71^), and are not discussed here. The secondaries thus produced, would undergo their own motion, deflections and scattering, and might be able to traverse the material from the point of their creation back to the top surface. In such an event, the secondaries could be emitted, if they had sufficient energy to transcend the work function barrier at the surface, as discussed in Eq. (3). By following a swarm of incident electrons and following the secondaries to determine if they might be ejected back out from the surface, the SEY can be numerically evaluated from the ratio of ejected particles to incident electrons.

There is a connection between the DFT calculations discussed in the preceding section and the kinetic Monte Carlo simulations given above. Basically, for following the transport of electrons in the host material with all of the on-going interactions, scattering, energy losses, deflections etc., one needs a set of parameters such the IMFP, energy loss function ($\frac{dE}{dR}$), details of selecting a target electron, and assignment of energies and trajectories after each binary collision. These parameters can be evaluated from a knowledge of the real- and imaginary-parts of the frequency-dependent permittivity of the host material. For self-consistency, these values are calculated from a first-principles DFT procedure. Details linking the permittivity to the IMF or Stopping Power are given later in Eqs. (4–7). Finally, the density of states (DOS) is also needed to select the target electron within the host with which a primary might interact and produce secondary offspring. The DOS provides information on all the possible electrons that the primary could potentially interact with and exchange its energy. Thus, there are a slew of available states, and the selection of the target electron from this distribution is based on using a random number as is standard procedure in stochastic discrete event simulations.

Upon undergoing an inelastic scattering event, primary electrons will lose an amount of energy, *∆E*, which can either be transferred to a shell electron or an electron in the Fermi sea of the material. After the collision, the primary particle will have energy *E − ∆E* remaining, while the secondary electron will either have an energy of *∆E* + *E*_*f*_ for case of energy transfer to an electron residing in the Fermi sea (where *E*_*f*_ denotes the Fermi energy), or it will have an energy *∆E − E*_*B*_, where *E*_*B*_ is an effective binding energy of the shell (for energy transferred to a core electron). The direction of the primary electron after the scattering event depends on the incident electrons momentum vector^84^ and the scattering angles as specified by *θ* and *ϕ* (in spherical co-ordinates). In our numerical procedure, the probability for a primary electron to interact with a target electron of energy *E*_*tar*_ in a uniform energy range *dE* was taken to be proportional to the fraction *f(E*_*tar*_*)* of available density of states *n(E)*, i.e., *f(E*_*tar*_*)* = *[n(E*_*tar*_*) dE]/[Σ*_*Ei*_* n(E*_*i*_*) dE]*. For a Monte Carlo implementation, the choice and result was in effect, then chosen by selecting a random number "*r*", and equation: *f(E*_*tar*_*)* = *r* as is the standard procedure for selection of a stochastic variable^85^. In the present calculations, the weighting of this ratio was determined by a random number in the range of [0,1] based on the Monte Carlo probabilistic simulation scheme^64,65^. For transport of electrons which is a random walk peppered by scattering events, the coordinates of secondary electrons depended on the location of the collisions, and their subsequent trajectories were determined based on angular outcomes of the discrete scattering events. The Monte Carlo simulated and tracked the positions, directions, and energies of all secondary and primary electrons. In depth discussion of these calculations, including the Monte Carlo based assignment of weightings are available in previous reports by our group^64,72^. In the present calculations, the bombardment of electrons in the Monte Carlo consisted of 5,000 electrons for computational efficiency. Also, particles would no longer be considered within the simulation after their energies had fallen below the thermal value of 1.5 *k*_*B*_*T*, with *k*_*B*_ being the Boltzmann constant and *T* the ambient temperature in the Kelvin scale.

Due to the surface potential barrier, it is necessary to consider the possibility of primary electrons being reflected at all energies within the Monte Carlo simulation. The quantum mechanical transmission feature was implemented in the Monte Carlo based on the approach outlined by Cohen-Tannoudji et al.^86^. The transmission probability for escape is given by:

3 $$T\left( {E,\theta } \right) = \left\{ {\begin{array}{*{20}l} {\frac{{4 \sqrt {1 - \left( {\frac{{U_{0} }}{{Ecos^{2} \theta }}} \right)} }}{{\left( {1~ + \sqrt {1 - \left( {\frac{{U_{0} }}{{Ecos^{2} \theta }}} \right)} } \right)^{2} }},\quad ~~for~\;Ecos^{2} \;\theta ~ > ~U_{0} ~~} \hfill \\ {0,~~\quad otherwise~~~} \hfill \\ \end{array} } \right.$$

where *θ* is the electron polar angle (normal to the surface), and *U*_*0*_ is the sum of the Fermi energy and work function of the surface. The angle of the emitted electrons (= *θ*^***′***^ ) is defined in terms of the polar angle of the scattering electron as: *√ E′ sinθ′* = *√ E sinθ*. As a consequence of crossing a surface/vacuum interface, the externally emitted electrons lose electron energy and have the energy *E*^***′***^ = *E − U*_*0*_.

Inelastic scattering of electrons within the host material has often been based on a continuous slowing down model in the literature, involving Joy and Luo’s generalization^87^ of Bethe’s stopping power formula^88,89^. In this context, the stopping power ($dE/dR$) is the energy ($E$) loss per unit length experienced by primary electrons along their traversal along a path of length $R$. More recently an improved technique, alluded to as the extended Mermin method for determining the inelastic mean free paths from measured optical energy loss functions was proposed^90^. It was shown that the resulting IMFPs for Cu and Mo based on the extended Mermin method, were in excellent agreement with the most reliable experimental measurements, which were not produced by other theoretical models. With inclusion of the dispersion relation, one can obtain the following inverse IMFP (${\lambda }^{-1}$) and stopping power (*dE/dR*) as given below [70 and references therein]:

4 $${\lambda }^{-1}=\frac{1}{\uppi {a}_{0}E}{\int }_{0}^{\infty }d\left(\hslash\upomega \right)\mathrm{Im}\left(\frac{-1}{\upvarepsilon \left(\upomega \right)}\right)\mathrm{ln}\left(\gamma \right)$$

and,

5 $$\frac{dE}{dR}=\frac{1}{\uppi {a}_{0}E}{\int }_{0}^{\infty }\hslash\upomega d\left(\hslash\upomega \right)\mathrm{Im}\left(\frac{-1}{\upvarepsilon \left(\upomega \right)}\right)\mathrm{ln}\left(\gamma \right)$$

where *a*_*0*_ is the Bohr radius, $\varepsilon (\omega )$ is the frequency-dependent dielectric tensor calculated using DFT methods^91^ and:

6 $$\gamma = \frac{2\surd (E - {E}_{F} - \hslash \omega )}{\surd (E) - \surd (E - 2\hslash \omega )}$$

It is well-known that the Mermin approach, overestimates the IMFP at lower energies (below 200 eV)^92^. The proposed method to correct the IMFP, dubbed the extended Mermin method, was introduced by Da et al*.*^90^. In the corrected IMFPs ${\lambda }_{e}$ is calculated from:

7 $${\lambda }_{e}= \lambda [1-{e}^{(-E/B)}]$$

where *λ* is the IMFP calculated from the Mermin approach and *E* is the electron kinetic energy. The parameter *B* is dependent on the material of the study and is used to correct the deviation from the Born approximation^90^. In this study the value of the *B* parameter was set to 24 eV for the calculation of the corrected IMFP for all systems as it was suggested by Da et al.^90^. It was shown that inclusion of the harmonic correction and modification of the Mermin model is essential for calculating the IMFP and stopping power^71^. Therefore, we used the same method in determining the IMFP and stopping power for all of the systems studied in this work.

## Results and discussion

Most available secondary emission studies have included or relied on polycrystalline samples. A first-principles study of polycrystalline systems is very complex and out of the scope of the work presented here. However, it has been shown^93,94^ that the crystal face with the lowest work function most closely resembles the polycrystalline surface. Since the (110) surface work function for FCC transition metals is known to be the lowest among the low-index surfaces^95^, we only study the (110) surface for the SEY modeling in the systems presented here. To study a crystal surface from first-principles, it is essential to determine the overall equilibrium positions of atoms. For the Cu system, the surface atomic concentrations of the “as received” sample in a study by Petit et al.^55^ were 24.3%, 40.6%, and 32.0% for Cu, C, and O, respectively. In order to model the exact experimental concentrations, one needs to consider a large supercell consisting of hundreds of atoms. Modeling such large supercells is computationally expensive. Therefore, we considered the closest concentration to experiment that could computationally be achieved while maintaining the physical aspects of the system. For that reason, we chose a 33.3% adsorbate (C and O) to substrate for the Cu system. Since there are no available experimental surface atomic concentrations for C and O to substrates in the case of Ag and Au, the same concentration as the Cu system was used in modelling the Ag and Au systems as well.

**Figure 1:**
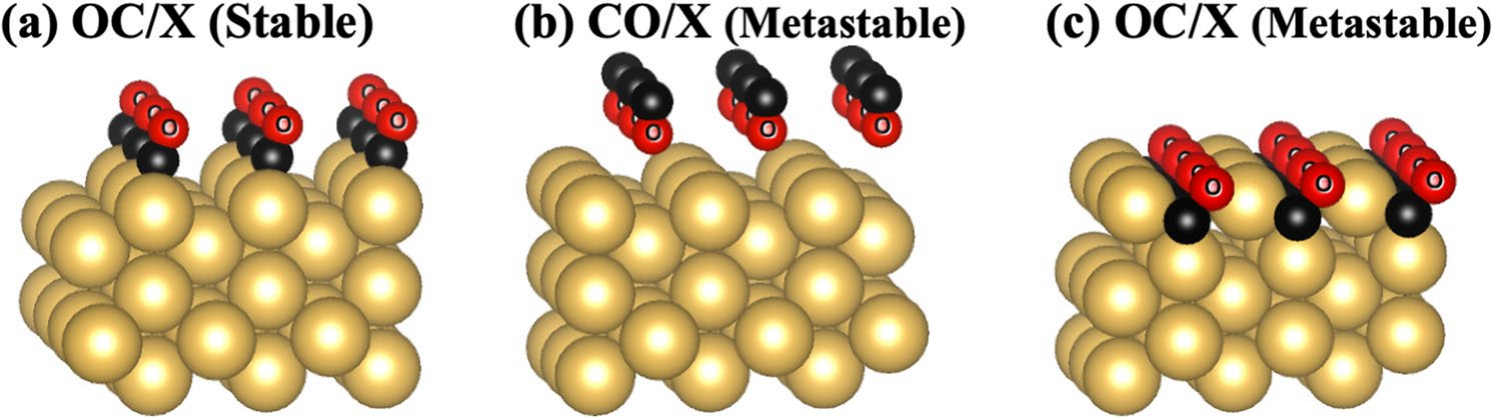
(color online). Crystal structures for: (**a**) stable OC/X, (**b**) metastable CO/X, and (**c**) the metastable OC/X. The big gold, small black, and small red spheres with small, inscribed circle indicate X (X = Cu, Ag, and Au), C, and O, respectively. Calculated energy difference between the OC/X (**a**) and CO/X (**b**) were − 0.491, − 0.121, and − 0.231 eV/atom for the Cu, Ag, and Au systems, respectively. The metastable OC/X (**c**) does not exist for Ag systems and has higher energy with respect to OC/X (**a**) 0.525 eV/atom for Cu and 0.608 eV/atom for Au systems.

Since the atomic positions and ordering of C and O atoms in these systems are not known, several configurations of C and O, were studied (Fig. 1). The OC/X system in Fig. 1a refers to the case where O is nearest to X (the host substrate) with C being furthest, while the situation is opposite for the CO/X system (Fig. 1b). The first-principles total energy calculations identify the OC/X configuration (Fig. 1a) as the most stable structure of all the systems. In all of the systems, the adsorbates OC and CO were positioned in a (1 × 1) orientation. For the most stable OC/X structure, the C atoms are closer to the X surface, occupying the short-bridge adsorption site, while the O atoms are more distant and sit atop the C atoms. There were two additional metastable structures identified and shown in Figs. 1b and c. When the O atoms are closer to the surface, CO/X (Fig. 1b), the O atoms occupy the long-bridge site with the C atoms positioned atop the O atoms. This metastable system has a higher energy with respect to the most stable OC/X structure. When the C atoms occupy the hollow adsorption site with the O atoms on top, another OC/X structure is created as shown in Fig. 1c. This metastable structure has a higher energy with respect to OC/X (Fig. 1a) and can only be formed for the Cu and Au systems. This abnormal behavior of Ag has also been observed for AgZn systems^96^ as well. Since Ag has a larger atomic size than Cu, the occupation of C in a similar site will cause an increase in the strain energy in the system resulting in an unstable structure. While Au has a similar size to Ag, the extended 5*d* orbitals of Au with respect to the 4*d* orbitals of Ag atoms allow Au to produce bonds with chemical energy strong enough to offset the additional strain energy component. In order to verify the stability and strength of the bond between substrate and adsorbate, the adsorption energy and cohesive (binding) energy were calculated for all systems. The adsorption energy characterizes the stability of the structure whereas the cohesive energy denotes the strength of the interaction^97^. A more negative adsorption energy corresponds to a more stable adsorbate substrate structure while a more positive cohesive energy indicates a stronger interaction between the adsorbate and substrate. The adsorption energy ${(\Delta H}_{ads})$ and cohesive (binding) energy $({\Delta H}_{coh}$) are calculated using,

8 $$\Delta H = {E}_{Sys} -{ E}_{Clean} - {E}_{molecule}^{CO}$$

where ${E}_{sys}$ is the energy of the carbon-oxygen layered surface and ${E}_{clean}$ is the energy of the clean surface. For adsorption energy ${(\Delta H}_{ads}),$ ${E}_{molecule}^{CO}$ is the calculated energy of an isolated CO molecule and for cohesive energy $({\Delta H}_{coh}$), ${E}_{molecule}^{CO}$ is the energy of a CO monolayer. The calculated CO molecule bond length values when adsorbed on the X surface are 1.32 Å, 1.18 Å, and 1.19 Å for the Cu, Ag, and Au surfaces, respectively. These values are in good agreement with the experimental bond length for an isolated CO molecule (1.128 Å^98^).

Calculated adsorption and cohesive energies are given in Table 1. The calculated CO/X cohesive energies were very small indicating that the formation of such a bond is unlikely. However, comparing the calculated value of the OC/X with available experimental measurements confirmed that first-principles calculations underestimate the cohesive energy by roughly 0.09 eV for Cu and Au systems. Therefore, inclusion of such a correction to the calculated cohesive energies of the CO/X systems results in metastable structures with cohesive energy of about 0.1 eV (Table 1). It is important to mention that the cohesive energy of the CO adsorption on transition metal surfaces is coverage-dependent^99^. In the case of low coverage, the binding (cohesive) energy will approach the adsorption energy. Therefore, one expects that the cohesive (binding) energies reported in Table 1 would be higher in the case of low CO adsorption coverage.

**Figure 2:**
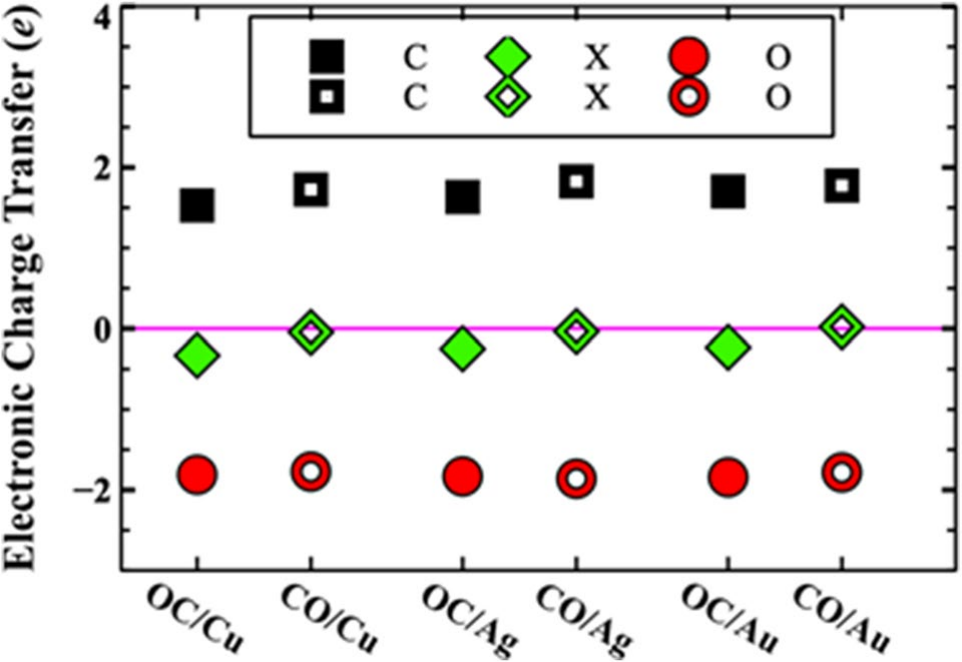
(color online). Electronic Charge Transfer ($e$) for all stable OC/X (solid markers) and metastable CO/X (hollow markers) systems. The pink line indicates the 0 charge transfer and is plotted to aid in distinguishing between gain and loss of charge(s).

For Cu, Ag, and Au (110) surfaces, the most stable configuration is the OC/X. The physical reason behind the stability is directly related to the nature of the bonding between C, O and X surface atoms. Bader charge analysis^100–103^ of C, O, and X surface atoms confirms a significant charge transfer to the O site mostly from C atoms and small charger transfer from the X surface atoms to the adsorbate (Fig. 2). Additionally, the amount of the charge transfer of the X surface atoms is smaller for the CO/X than the OC/X system (Fig. 2) resulting in a weak bond in agreement with calculated cohesive energy (Table 1). The small cohesive energies and charge transfer between the CO adsorbate and X surface, indicates a strong interaction between the C and O atoms and weak interactions with the X surface atoms for all structures. Indeed, calculated binding energy of CO molecule was 6.60 eV which is comparable to the theoretical value of 7.45 eV^104^ and is much larger than cohesive energy of OC and X surface for all systems.

**Table 1:** Calculated adsorption energies $\Delta {H}_{ads}$ (eV/atom), cohesive energies $\Delta {H}_{coh}$ (eV/atom), work functions $\varphi$ (eV), normal component of the dipole moment *p*_*⊥*_ (eÅ), and Fermi level energy *E*_*F*_ (eV) for the transition metal substrates X (X = Cu, Ag, and Au), CO/X, and OC/X. Experimental work functions are provided for comparison.

110 Surface	$\Delta {H}_{ads}$(eV/atom)	$\Delta {H}_{coh}$(eV/atom)	${\varphi }_{cal}$(eV)	${p}_{\perp }$(eÅ)	*E*_*F*_ (eV)
Cu	n/a	n/a	4.40 (4.48 ^a^)	n/a	8.91
OC/Cu	− 2.795	0.50 (0.59 (^b,c^))	6.07	0.1135	10.66
CO/Cu	− 2.304	0.01	4.95	0.0391	10.12
Ag	n/a	n/a	4.16 (4.14 ^d^)	n/a	7.26
OC/Ag	− 2.546	0.12	5.28	0.1025	10.18
CO/Ag	− 2.425	− 0.01	4.54	0.0260	10.31
Au	n/a	n/a	4.98 (5.12 ^e^)	n/a	7.25
OC/Au	− 2.659	0.26 ($\le$ 0.35 f.)	5.88	0.0851	11.89
CO/Au	− 2.428	0.03	4.97	− 0.0036	9.81

^a^Reference^105^, ^b^Reference^106^, ^c^Reference^107^, ^d^Reference^108^, ^e^Reference^109^, ^f^Reference^110^.

The calculated work function and the Fermi level energy for all of the systems were compared with available experimental data, and are provided in Table 1. One can see some interesting trends in work function: (i) The work function is reducing for all systems when CO added to the surface, (ii) The change of the work function with respect to their clean surfaces are largest for Cu and smallest for Au. In order to explain these trends, one should consider the effect of the created electric dipole moment as a result of the charge transfer between the C and O atoms (Fig. 2). The normal component of the dipole moments (*p*_⊥_) was calculated for all OC/X systems and are given in Table 1. From Table 1, one can see a direct correlation between the work function and the normal component of dipole moment. The role of the dipole moment in influencing the work function is well known^111^, and was invoked to explain the role of coatings on field emitters^63^. The larger the dipole moment, the greater the work function change. Interestingly, for the CO/Au system the work function is slightly smaller than the clean Au surface. This is in agreement with the reversal of the dipole moment direction with respect to some of the other systems. Furthermore, the calculated work function values for all three clean metals in Table 1 are in good agreement with published data. The work functions, Fermi energies, and dielectric functions were used as input parameters to calculate the inelastic mean free paths (IMFP) and secondary electron emissions (SEY). For the rest of this work, we will only study and discuss the most stable OC/X structure given in Fig. 1a.

**Figure 3:**
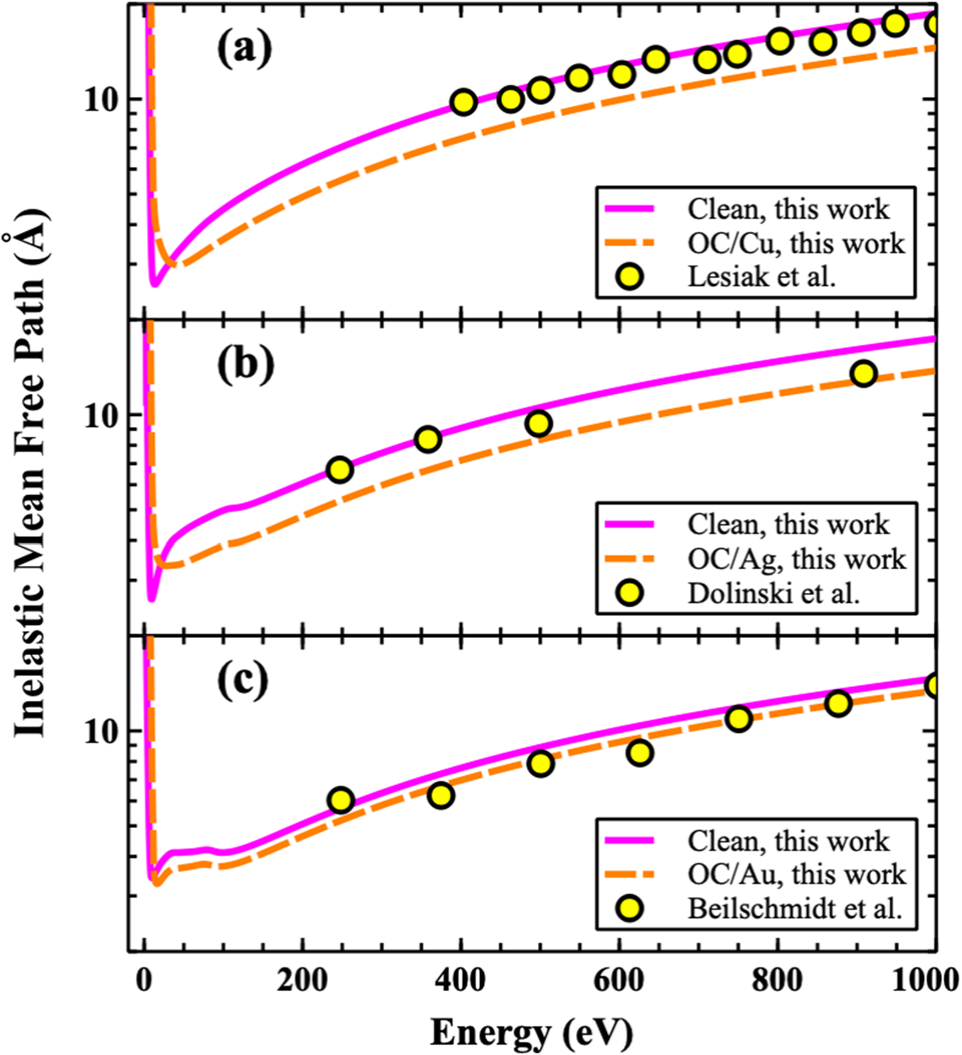
(color online). The calculated IMFP (using Eqs. 4 and 7) results for: (**a**) Cu, (**b**) Ag, and (**c**) Au systems. Experimental results are also provided for comparison including Lesiak et al.^112^, Dolinski et al.^113^, and Beilschmidt et al.^114^ for Cu, Ag, and Au, respectively.

The results of the IMFP for the Cu, Au, and Ag (110) surfaces are shown in Fig. 3. As one can see from the figures, the calculated IMFP’s show very good agreement with experimental measurements^112–114^. The addition of the OC layers to all of the clean surfaces causes a decrease in the IMFP across the entire energy spectrum (Fig. 3). The physical reason of the IMFP decrease is directly related to change of the density of states (bonding between C, O and X) that will be discussed shortly.

**Figure 4:**
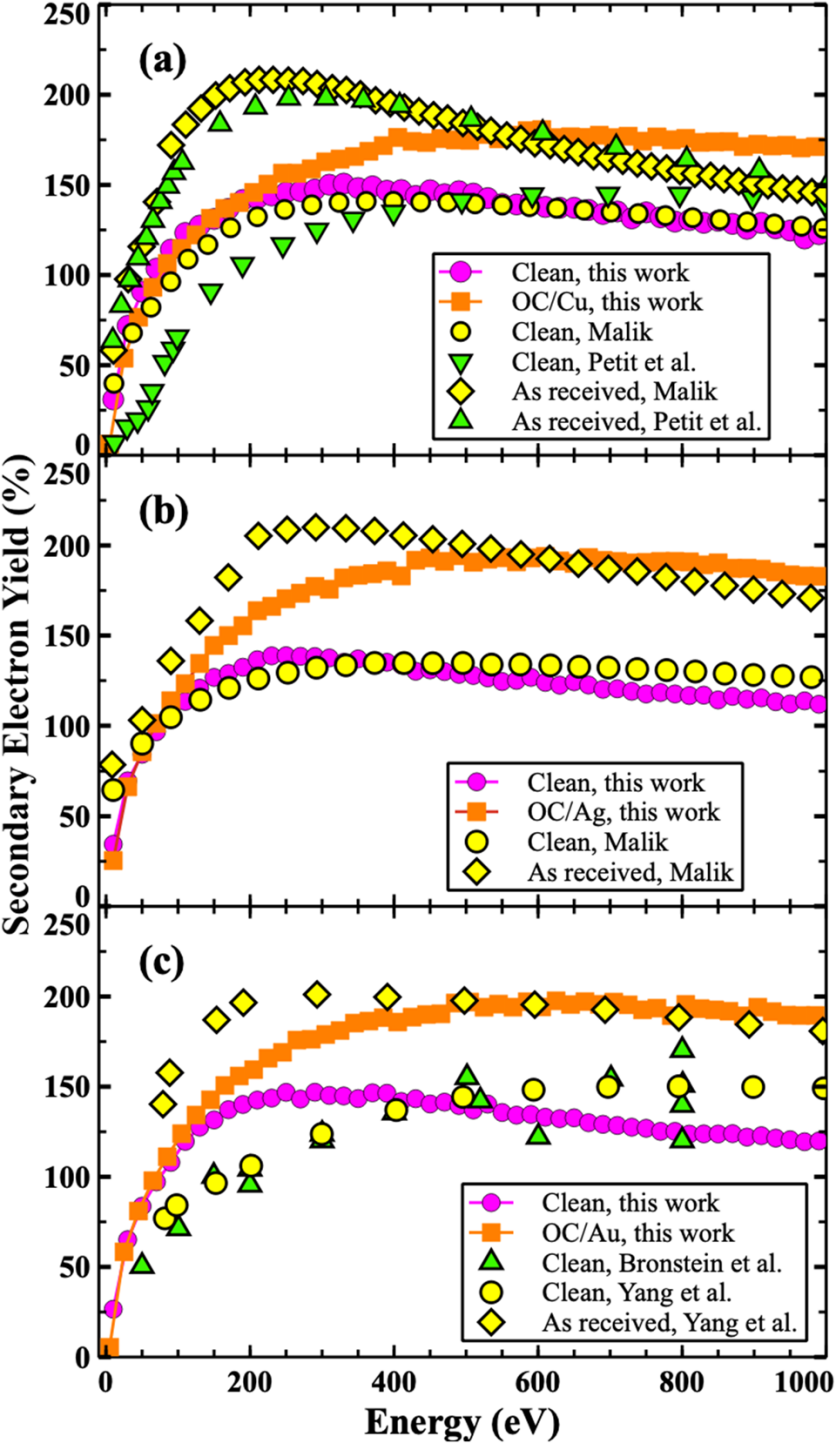
(color online). Secondary electron yield for three system: (**a**) Cu, (**b**) Ag, and (**c**) Au systems. Experimental measurements are also provided for comparison including the results from Petit et al.^55^, Yang et al.^60^, Malik^61^, and Bronstein et al.^115^.

The SEY calculations of the clean and OC/X structures are shown in Fig. 4 and are compared with available experimental results^55,60,61,115^. An overall agreement between the simulated and experimental measurements can be seen in all of the plots of Fig. 4. Also, the presence of the OC adsorbate is predicted to significantly increase the SEY for all of the systems. The origin of the slight differences is likely related to the use of polycrystalline samples^55,60,61^ instead of the high symmetric surfaces used in this study. Additionally, minor deviations could also stem from the presence of other impurities in the actual experimental samples and their possible two-dimensional distributions along the surface. Variations in lateral distribution under experimental conditions such as partial or nonuniform coverage of the surface can also influence the SEY outcomes. The coverage would affect the work function at the surface. If there were parts of the surface that were bare, while others had adsorbates, the works functions would then vary laterally due to the heterogeneity of the surface. As a result, the SEY would have a patch-like variation, with lateral changes mimicking the lateral detail of the coverage. The net behavior would be a weighted sum of all such individual SEY emission from elemental surface patches. In addition, even if the entire surface was covered but the thickness varied, the work function would vary laterally, leading to a similar result. Furthermore, the “as received” sample (mentioned in our referenced example) could have had other impurities in them such as N, H_2_O, NO, CO_2_ etc. Moreover, the present calculations assumed a smooth surface with no roughness and an ideal uniform-distribution of OC at the top surface, which could be different from the actual samples tested. For example, roughened surfaces could have mini-terraces or numerous small tilts. For a surface having a small angular tilt *α*, the threshold condition in Eq. (3) would change to: *E *cos^*2*^*(θ-α)* > *U*_*0*_. As a collective result, smaller energies would be allowed to pass over the threshold, and the overall SEY value would increase due to surface tilts or corrugation, as in the measured data. Despite all of the possible variability, however, the present calculations do demonstrate reasonable agreement with data, and also show that the addition of O and C adsorbates at the surface could be a source of SEY enhancements. The results also suggest that cleaning the sample surfaces to get rid of the O and C adsorbates might be beneficial for SEY lowering. Furthermore in Fig. 4, the strongest deviation between clean material and the SEY predictions with and O and C absorbates included, is for silver and gold. While the trends are reassuring, one cannot fully explain the SEY increases for the cases with surface absorbates based on work function (tabulated in Table 1) changes alone. This aspect is probed further, and the role of the material density of states appropriately included in the discussion.

As evident from Table 1, the work function typically is lower for the clean surfaces. This implies that it would not be as easy for secondaries with a lower energy level to be ejected out if the surface was not clean. With contamination, the electron ejection, and hence, contribution to the SEY is pushed to higher energies, and results in a rightward shift of the SEY curves of Fig. 4. In addition, as shown in Fig. 3, the inelastic mean free paths for electron is slightly larger for clean surfaces. A higher value leads to deeper penetration of the primary electrons, making it more difficult for the secondaries to traverse and successfully make it to all the way back to the surface. Consequently, one expects a smaller SEY strength for cleaner material. Finally, the role of the Stopping Power on the SEY also has to be considered. The energy dependence of the Stopping Power (SP) exhibits a local maxima, and then reduces monotonically with energy. More importantly, though not shown, the SP for pure material is slightly reduced in comparison to CO/X material. This implies that the entering electrons with the highest energy at or near the surface would have lower SP values and hence, the creation would occur deeper in the material. Finally, inclusion of the harmonic correction for the SP calculations, also contributes to a flattening of the SEY. This was also shown in one of our recent reports of surface hydrogen modification to the SEY in Nickel^71^. Thus, the overall effect is an SEY characteristics for OC/X that is larger with a greater spread towards higher energies relative to the characteristics from pure material.

**Figure 5:**
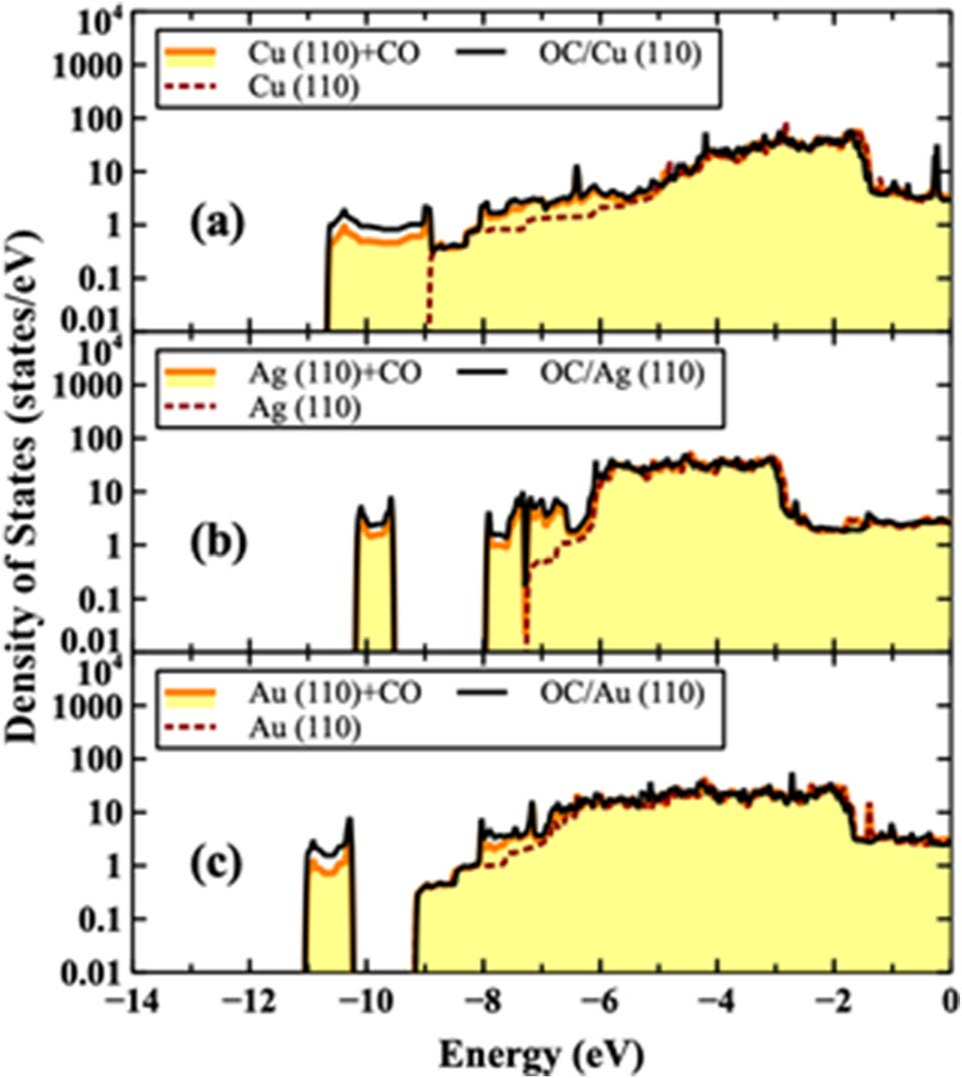
(color). Calculated total densities of states for X (110) clean surface (dashed), X (110) + CO (shaded), and OC/X (110) (solid line) for all systems. The partial density of the CO can be obtained by the difference between the X (110) + CO and the X (110) densities. The Fermi level energy is located at 0 eV.

In order to understand the increase of the SEY, it is essential to carefully study the role of the OC adsorbate in these systems. Studying the total and partial densities of states gives additional insight into the nature of the bonding between elements. As it was discussed previously, based on the Bader charge analysis and cohesive energy calculations, there is a weak interaction between CO and the X surface (X = Cu, Ag, or Au) and a strong binding between C and O atoms. One can expect that such interactions can also be realized from analyzing the total density of states (TDOS). In order to qualitatively verify the strength of those interactions, the TDOS for X (110) clean.

surface, X (110) + CO, and OC/X (110) for all systems were calculated and depicted in Fig. 5. As can be seen in Fig. 5, there is no significant change between the TDOS of the X (110) + CO and the OC/X surfaces, indicating that CO is weakly bounded to the X surface. The analysis of the partial density of states (PDOS) of C and O atoms, and comparing the TDOS, confirm the strong bonding between C and O atoms (specifically, the sharp densities around − 10 eV for all systems).

Since there is no significant bonding between CO and X (X = Cu, Ag, or Au) atoms, there is no pronounced changes of TDOS for Cu, Ag, and Au atoms at higher energies (above − 6 eV), and C and O densities are simply added to the TDOS of each system, resulting in further extending the TDOS at lower energies.

Based on results of the TDOS analysis, one can explain the changes in the inelastic mean free paths and SEY for all of the systems. The change of the TDOS has a direct impact on the electronic probability distribution (EPD) of the system. The EPD was used as a metric in the Monte Carlo simulations for the acceptance or rejection of secondary electron creation^72^ and is defined by the following:

9 $$EPD\left(E\right)= \frac{\underset{{E}_{min}}{\overset{E}{\int }}f\left(E\right) n\left(E\right) dE }{\underset{{E}_{min}}{\overset{E_{F}}{\int }}f\left(E\right) n\left(E\right) dE}$$

where *f(E)* is the Fermi–Dirac distribution, *n(E)* is the total density of states, *E*_*F*_ is the Fermi energy of the system, and ${E}_{min}$ is the minimum energy of the calculated TDOS.

**Figure 6:**
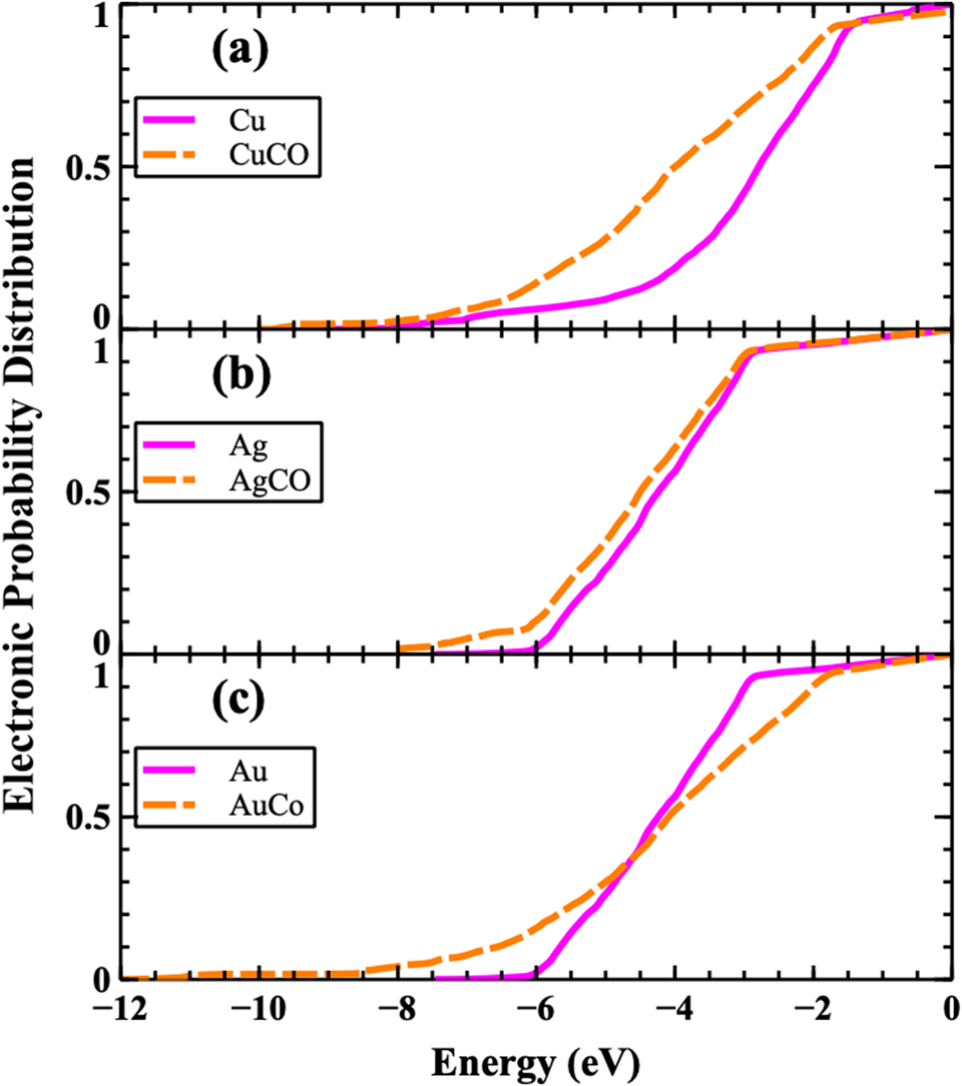
(color online). Electronic probability distribution with and without the OC absorbates for the: (**a**) Cu, (**b**) Ag, and (**c**) Au systems. The Fermi level energy is again taken to be at 0 eV.

In Fig. 6, the calculated EPD for all systems are provided with and without the absorbates. For the Cu system (Fig. 6a), there is a significant increase of the probability in the range of − 5 to − 2 eV. For Ag and Au systems (Figs. 6b and c), the EPD is seen to increase and extend to lower energies in the presence of absorbates. This increase of EPD causes a corresponding increase in the inelastic scattering events between electrons and also reductions in inelastic mean free paths (Fig. 3). Interestingly, it may also be mentioned that in spite of the small changes in EPD and IMFP of Au, the OC/Au SEY is comparable to the other systems. This is directly related to the smaller change in work function of Au ($\Delta \varphi$ = 0.90 eV) with respect to Cu ($\Delta \varphi$ = 1.67 eV) and Ag ($\Delta \varphi$ = 1.12 eV) systems. For all the systems studied, despite an increase of the work function, increases in the density of electrons combined with reductions of mean free path (higher collision probability) cause an increase of SEY. Therefore, by removing and cleaning the C and/or O atoms from such samples, the SEY is predicted to be significantly reduced.

## Summarizing conclusion

Using a combined first-principles and Monte-Carlo method, the formation of OC monolayer on (110) surfaces of Cu, Ag, and Au materials was studied. The most stable structure was identified as OC/X. The work function for all structures were calculated, compared with available experimental results, and shown to match reported data for all three pure metals. The dielectric function and total density of states were also calculated for each system and used as input parameters for the Monte Carlo simulation of electron transport in the host material. The IMFP and SEY between 0 and 1000 eV for clean and OC/X surfaces were obtained and compared with available experimental measurements. Reasonably close agreements were obtained. It was shown that the presence of OC adsorbate increased the energy-dependent SEY values comparable to the “as received” samples reported in the literature for each system. The analysis of the total density of states of all systems confirmed a strong bond between C and O. This was shown using the TDOS, where the inclusion of the C and O atoms did not change the density at higher energies but simply added to the density. So, while the work function can be a major controlling parameter for SEY in many systems, it was shown that in the OC/X system, that it is the change of electron density that actually leads to an increase of the SEY with respect to clean surface in the present case. It was concluded that the removal of the OC surface layer though an appropriate cleaning procedure would help decrease the SEY in all systems as has been observed in experiments.

Thus, in summary, a coupled method involving first-principles calculations for the material parameters and a kinetic Monte Carlo for electron transport in host materials was used to compute the SEY. In particular, the influence of absorbates at the surface on possible modifications to the SEY were probed. Though emission from Cu, Ag, and Au were evaluated, and a mix of O and C atom at the surface were considered as representative contaminants, the method is general and could be used to investigate other emitter materials and/or surface absorbates as well. Other possible scenarios could include the formation of graphitic carbon during irradiation or the presence of carbon-free oxidized (Cu_2_O) copper^55^, or laser processing in nitrogen-rich environments that can reportedly reduce SEY^53^. The role of water vapor could be another situation^61,116^, as would changes in SEY with surface oxide layer thickness. Variations in surface coverage or stepped layers would be more arduous extensions involving two-dimensional lateral variations in the surface potentials, work functions, position dependent man free paths etc. It would also be interesting to obtain improved analytic fits to energy-dependent SEY characteristics starting from the Vaughan’s expression^57^ as was reported by Ludwick et al.^117^, due to the presence of such adsorbates from the standpoint of computational convenience.

## Data Availability

The data that support the findings of this study are available from the corresponding author upon reasonable request.
